# Looking for Resistance to Soft Rot Disease of Potatoes Facing Environmental Hypoxia

**DOI:** 10.3390/ijms25073757

**Published:** 2024-03-28

**Authors:** Tomasz Maciag, Edmund Kozieł, Katarzyna Otulak-Kozieł, Sylwia Jafra, Robert Czajkowski

**Affiliations:** 1 Department of Botany, Institute of Biology, Warsaw University of Life Sciences—SGGW, Nowoursynowska Street 159, 02-776 Warsaw, Poland; katarzyna_otulak@sggw.edu.pl; 2 Laboratory of Plant Microbiology, Intercollegiate Faculty of Biotechnology UG and MUG, University of Gdansk, Antoniego Abrahama Street 58, 80-307 Gdansk, Poland; sylwia.jafra@ug.edu.pl; 3 Laboratory of Biologically Active Compounds, Intercollegiate Faculty of Biotechnology UG and MUG, University of Gdansk, Antoniego Abrahama Street 58, 80-307 Gdansk, Poland; robert.czajkowski@biotech.ug.edu.pl

**Keywords:** soft rot, potato, *Dickeya*, *Pectobacterium*, *Erwinia*, waterlogging

## Abstract

Plants are exposed to various stressors, including pathogens, requiring specific environmental conditions to provoke/induce plant disease. This phenomenon is called the “disease triangle” and is directly connected with a particular plant–pathogen interaction. Only a virulent pathogen interacting with a susceptible plant cultivar will lead to disease under specific environmental conditions. This may seem difficult to accomplish, but soft rot *Pectobacteriaceae* (SRPs) is a group virulent of pathogenic bacteria with a broad host range. Additionally, waterlogging (and, resulting from it, hypoxia), which is becoming a frequent problem in farming, is a favoring condition for this group of pathogens. Waterlogging by itself is an important source of abiotic stress for plants due to lowered gas exchange. Therefore, plants have evolved an ethylene-based system for hypoxia sensing. Plant response is coordinated by hormonal changes which induce metabolic and physiological adjustment to the environmental conditions. Wetland species such as rice (*Oryza sativa* L.), and bittersweet nightshade (*Solanum dulcamara* L.) have developed adaptations enabling them to withstand prolonged periods of decreased oxygen availability. On the other hand, potato (*Solanum tuberosum* L.), although able to sense and response to hypoxia, is sensitive to this environmental stress. This situation is exploited by SRPs which in response to hypoxia induce the production of virulence factors with the use of cyclic diguanylate (c-di-GMP). Potato tubers in turn reduce their defenses to preserve energy to prevent the negative effects of reactive oxygen species and acidification, making them prone to soft rot disease. To reduce the losses caused by the soft rot disease we need sensitive and reliable methods for the detection of the pathogens, to isolate infected plant material. However, due to the high prevalence of SRPs in the environment, we also need to create new potato varieties more resistant to the disease. To reach that goal, we can look to wild potatoes and other *Solanum* species for mechanisms of resistance to waterlogging. Potato resistance can also be aided by beneficial microorganisms which can induce the plant’s natural defenses to bacterial infections but also waterlogging. However, most of the known plant-beneficial microorganisms suffer from hypoxia and can be outcompeted by plant pathogens. Therefore, it is important to look for microorganisms that can withstand hypoxia or alleviate its effects on the plant, e.g., by improving soil structure. Therefore, this review aims to present crucial elements of potato response to hypoxia and SRP infection and future outlooks for the prevention of soft rot disease considering the influence of environmental conditions.

## 1. Introduction

Throughout their lives, plants are exposed to various kinds of stress, which can be divided into biotic and abiotic. Biotic stress is caused by living organisms (such as animals, fungi, bacteria, and other plants), viruses, and viroids. Meanwhile, physical or chemical environmental factors cause abiotic stress [1]. The influence of these factors on plant health is usually analyzed independently. However, they frequently co-occur and, currently, we observe a growing number of studies dedicated to the influence of joint stress factors on plants and their response to them [2]. Abiotic stress affects plants and their pathogens, forcing plants to adjust their response to both factors [3]. Plant pathogens often cannot induce disease symptoms in conditions favorable for their host. Therefore, developing the disease requires a so-called “disease triangle” composed of three elements: virulent pathogens, susceptible plants, and appropriate environmental conditions [4].

Environmental stress, such as hypoxia (a state of lowered oxygen availability (1–5%)), can favor plant susceptibility to certain diseases [5]. Hypoxia inhibits plant aerobic respiration, leading to decreased metabolism, slowed growth, and lowered resistance to other stress factors, such as necrotrophic pathogens [6] or herbivorous animal invasion [7]. Hypoxia, as a stressor, is mostly induced by waterlogging [8]. The temporal rise in the water level leads to the submerging of the main part of the roots and/or aerial plant parts [9]. The soil air pockets are filled with water, additionally oxygen, as a nonpolar gas, poorly dissolves in water, leading to decreased oxygen mobility and availability for the submerged parts of the plant [10]. The result is a drastic change in plant metabolism, making the plants more susceptible to other environmental factors [9]. Above that, waterlogging causes soil structural rearrangement due to the high density of water leading to soil compaction. In the compact and deprived-of-oxygen soil, toxic microbial metabolites accumulate, and decreased redox potential leads to a drop in the soil pH and an increased amount of toxic metal ions [11]. Therefore, waterlogging can cause huge economic losses in agriculture and it is estimated that on average it reduces yield by 33%, which is expected to largely increase due to climate change and the deterioration of soil structure [12].

However, hypoxia can also be a more local physiological state for certain tissues or cells. For example, low oxygen content in meristematic tissues prevents cells from differentiation [13]. Decreased oxygen penetration into nodules, structures housing plant-beneficial *Rhizobia* and *Sinorhizobia*, causes plants to protects their nitrogenase from oxidization [14]. Also, thick and water-loaded structures such as tubers [15] or fruits [16] need to adapt to lower oxygen availability by decreasing the primary metabolism rate in the central part of the structure [17].

Due to low oxygen penetration to plant tissues, only the external part of the plants have a high metabolic activity [18]. The epidermis and subepidermal parts of plants’ organs have the best access to atmospheric oxygen and, therefore, the proper energetic potential to induce plant defenses, including secondary metabolism, and defend themselves against pathogen invasion [19]. But when the epidermis barrier is broken [20] or when oxygen diffusion is blocked, epidermal cells decrease their metabolism rate [17] and then the pathogens have access to less defended internal parts [21].

**Figure 1:**
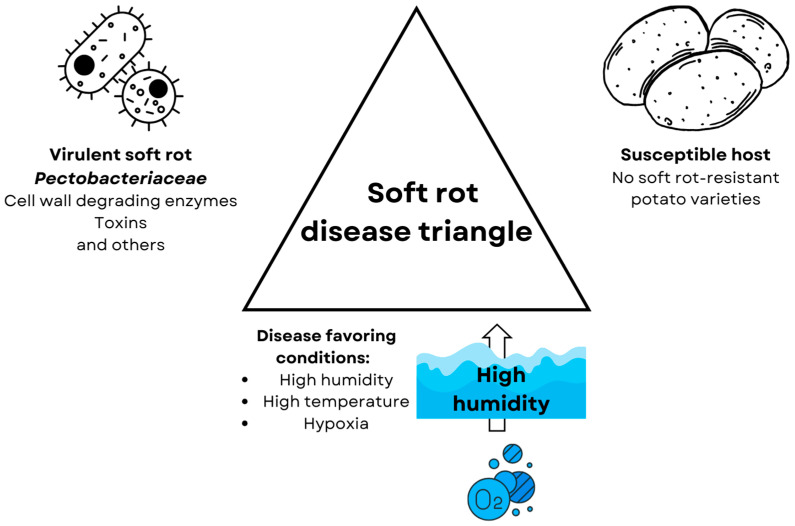
The disease triangle for soft rot disease. All three components are necessary for the induction of soft rot disease. (1) Virulent strain of soft rot *Pectobacteriaceae*, whose virulence depends greatly on the production of cell wall degrading enzymes, but also other virulence factors such as the production of toxins [31], like necrose inducing protein (Nip) [32] and the type 4 [33] and 6 secretion systems [34]. (2) Susceptible hosts such as potatoes, currently there are no potato varieties resistant to soft rot disease [35,36]. (3) Disease-favoring conditions which are high humidity and temperature and, resulting from high humidity, hypoxia [37].

Waterlogging, however, does not only directly influence plant metabolism but also its inhabiting microorganisms, including pathogens. For some pathogens, the decreased oxygen availability can restrain their survival (e.g., *Collybia fusipes* [22]) and pathogenicity (e.g., *Fusarium graminearum* [23]), but others can benefit from it [6]. One of the most important bacterial plant pathogens, soft rot *Pectobacteriaceae* (SRP) [24], benefits from hypoxia using its adaptations [25] and the lowered defenses of the plant [26]. These bacteria cause soft rot disease in fruits and vegetables. Soft rot is one of the most economically important diseases affecting potatoes (*Solanum tuberosum* L.)—the utmost widely consumed vegetable [27]. SRPs enter the tubers through wounds or lenticels and, depending on the environmental conditions, either form latent infection or multiply and penetrate deeper into the plant tissues leading to the development of the disease [28]. Latently infected tubers (without any symptoms) are the most common source of these pathogens [29]. Latent infections lead to disease development under disease-favoring conditions: elevated temperature, high humidity, and low oxygen availability [30]. Summarizing according to the “disease triangle” concept [4], soft rot *Pectobacteriace* leads to the development of the soft rot disease in high-temperature and -humidity conditions in susceptible hosts such as potatoes by the production of virulence factors which are mainly plant cell wall degrading enzymes but also toxins [31], such as necrosis-inducing virulence protein (Nip) [32], but also type 4 secretion system (T4SS) [33], and type 6 secretion system (T6SS) [34] (Figure 1).

So far, attempts to create soft rot-resistant potato varieties have not succeeded [35,36]. It is proposed that the climate catastrophe will further increase the losses caused by SRP [38]. Therefore, it is imperative to quickly develop new strategies to protect potatoes from these pathogens. *Solanum* L. plant species that are better adapted to waterlogging can be a good source of soft rot disease resistance [39,40,41].

## 2. Plant Response to Hypoxia

### 2.1. Hypoxia Sensing

**Figure 2:**
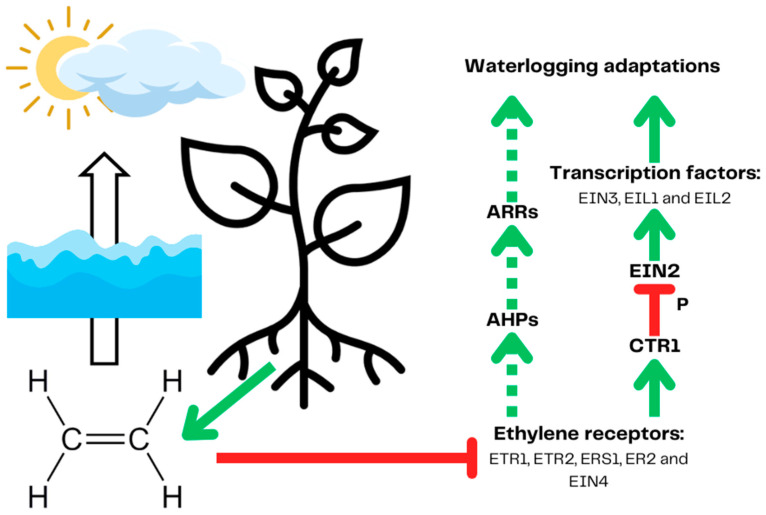
Plant ethylene signaling leads to the expression of genes, leading to waterlogging adaptations. Constantly produced ethylene is trapped in submerged tissues due to the low diffusion of nonpolar gases in water and cannot escape into the atmosphere. Ethylene is then bound by ethylene receptors, inhibiting their activity. In the canonical pathway, ethylene receptors bind ethylene preventing them from activating constitutive triple response 1 (CTR1). Inactive CTR1 kinase no longer phosphorylates ethylene-insensitive 2 (EIN2), leading to its activation and following the activation of hypoxia transcription factors. The non-canonical pathway (marked by a dotted line) starts with a suppressed ETR1 receptor, which no longer activates CTR1 phosphorylates Arabidopsis histidine-containing phosphotransfer proteins (AHPs) (linking the ethylene sensing with cytokine signaling), which in turn activate Arabidopsis response regulators (ARR) by phosphotransfer. ARRs regulate the expression of waterlogging response genes. Prepared based on [43,45]. The red arrows demonstrate inhibition and the green arrows represent induction.

To survive short-term hypoxia, plants developed a signaling system to adapt to lower oxygen availability [42]. The system comprises hypoxia detection mechanisms and signaling systems cooperating with plant phytohormones, which can coordinate plant metabolic and morphological adjustments to lower oxygen availability. The presented signaling pathways are established mainly on the Arabidopsis (*Arabidopsis thaliana* (L.) Heynh.) model unless stated otherwise. The key element for hypoxia detection and signaling is ethylene, which is produced by all plant tissues but quickly escapes into the atmosphere [43]. Waterlogging disrupts the diffusion of nonpolar ethylene, accumulating in the plant’s oxygen-deprived parts (Figure 2) [43]. Ethylene is then bound by its receptors, inhibiting its ability to deactivate (phosphorylate) ethylene-insensitive 2 (EIN2) by constitutive triple response 1 (CTR1). Unphosphorylated EIN2 activates hypoxia transcription factors such as EIN3 and EIN3-like 1 and 2 (EIL1, EIL2) in the canonical pathway [44]. It has been proven that the ethylene signaling pathway is activated during hypoxia in *Solanaceae* but it does not lead to the activation of hypoxia response in waterlogging-susceptible potato varieties nor does it in tomato (*Solanum lycopersicum* L.). This implies that the differences responsible for different tolerance to waterlogging lie downstream from the explained signaling pathway [39]. The presence of an as yet not fully described non-canonical pathway connecting ethylene signaling with abscisic acid (ABA), a phytohormone, enables tighter regulation of plant response to hypoxia [45]. ABA, downregulated in soybean (*Glycine max* (L.) Merr.) during waterlogging, impairs the formation of adventitious roots and aerenchyma, positively affecting hypoxia tolerance [46].

On the other hand, ABA promotes stomata closure and decreases hypoxia-caused senescence [47]. Another type of phytohormone, auxins, are upregulated and act antagonistically on ABA, they also promote aerenchyma and adventitious root formation in bittersweet nightshade (*Solanum dulcamara* L.) [48]. The involvement of auxins and ABA in the glucose–TOR signaling pathway [49] and ABA’s role in the non-canonical pathway of ethylene signaling [45] integrates nutrient status sensing with oxygen availability. The involvement of ABA in the non-canonical pathway of ethylene signaling integrates an energetic status to respond to waterlogging [45]. Meanwhile, an increased gibberellin concentration significantly causes plant internode elongation in deepwater rice (*Oryza sativa* L.), helping plants’ shoots and/or roots extend beyond the water level to reach oxygen [50]. Waterlogging can also lead to the accumulation of salicylic acid (SA), which is a major plant hormone responsible for resistance to abiotic stress and biotic stress caused by biotrophic pathogens [51]. SA promotes apoptosis, which is necessary for aerenchyma formation and the development of adventitious roots in Soybean [52]. Jasmonic acid (JA)—an SA antagonist, another major plant hormone, is responsible for plant resistance against herbivorous animals (mainly insects and arachnids) and necrotrophic pathogens [53]. For example, in citruses (*Citrus*), hypoxia leads to elevated JA in leaves but decreased JA levels in roots [54]. In waterlogging-sensitive cucumber (*Cucumis sativus* L.), the concentration of JA in hypocotyl was upregulated but downregulated in waterlogging-resistant lines [55]. This may result from the formation of adventitious root from hypocotyl. JA can positively influence waterlogging tolerance by increasing the concentration of ethylene in conifers [56] but, on the other hand, suppressing root growth and SA activity in soybeans [57]. Therefore, the concentration gradient of JA from shoots to the roots positively affects waterlogging tolerance. Also, brassinosteroids can positively resist waterlogging by promoting adventitious root development, hypocotyl expansion, and loosening in cucumber [58]. Relatively limited studies compared to other plant hormones are dedicated to melatonin’s effects on waterlogging [9,59]. It has been reported that melatonin can increase waterlogging tolerance by alleviating the negative impact of reactive oxygen species in Siberian crab apples (*Malus baccata* Borkh.) [60]. Alternatively, Zheng et al., 2017 [60] reported that melatonin reduces ethylene concentration, suggesting an antagonistic interaction, but this requires further study.

### 2.2. Adaptation to Hypoxia

While ethylene and energetic status are hypoxia sensors, ethylene, and plant hormones allow plants to integrate the response between different plant parts and tissues. To adapt their cellular metabolism to hypoxia, plants rely on ethylene response factors VII (ERF VII), which activate the transcription of hypoxia response genes. Plant cysteine oxidases oxidize the N-terminus cysteine residue of this protein, leading to ERF VII proteasomal degradation. During hypoxia, stabilized ERF VIIs activate the transcription of hypoxia-responsive genes (HRGs) [61]. ERF VIIs can integrate ethylene sensing and energetic status to induce the expression of the hypoxia response genes [62]. ERF VIIs signaling allows plants to induce anaerobic respiration before the tricarboxylic acid cycle deprives the tissues of remaining oxygen in the pea (*Pisum sativum* L.) [63]. However, alcohol fermentation is much less energetically efficient and leads to root carbon deprivation. In response to the high demand for organic carbon in flooded roots, carbohydrates are transported to the roots of ash (*Fraxinus*) [64]. The increased demand for organic carbon can never be met by photosynthesis, since its rate is downregulated during hypoxia. Due to stomata closure and lowered gas exchange, photosynthesis is less efficient in cotton (*Gossypium hirsutum* L.) [65].

Hypoxia also leads to the accumulation of reactive oxygen species by the mitochondrial electron transport chain, causing chlorophyll degradation and further decreasing the plant’s photosynthetic potential [66,67]. To tackle that problem, plants can recruit stored lipids for energy, signaling, and protection from toxic products of anaerobic respiration [68]. Due to the multiple ways environmental hypoxia can negatively influence plant development, the best-adapted waterlogging plants such as rice minimize their metabolic rate during stressful periods [69]. Wetland plants, such as *Carex* L., however, need anatomic adaptations for more efficient oxygen transportation [70].

Although lowered metabolism can help to withstand short-time hypoxia, wetland crops including rice need to maintain an efficient metabolism inside their flooded roots. This can only be achieved by root architectural changes [71] and metabolic adaptation [72]. One of the water plants’ adaptations for decreased oxygen availability is the development of internal ventilation [73]. Cortex cells deprived of oxygen die and degrade, forming natural cavities—resulting in aerenchyma formation. Aerenchyma can foster oxygen transportation and increase the adsorption area. Depending on the species, high water levels can lead to aerenchyma formation (e.g., wheat (*Triticum aestivum* L.) and corn (*Zea mays* subsp. *mays* L.) and an increase in its volume (e.g., rice) [71]. Rice exposed to waterlogging on its aerial (green) parts develops thin layers of gas film on its leaf surface, enabling gas exchange during submerging [74].

Another important adaptation is the development of a radial oxygen loss (ROL) barrier in many plant species. ROL limits the gas exchange to the root tips, decreasing oxygen loss in the upper part of the roots while still enabling the uptake of nutrients [75] In addition, cereals increase their roots’ cortex-to-stale ratio and produce fewer and shorter lateral roots to decrease oxygen consumption and increase soil penetration [71]. To penetrate the topsoil levels richer in oxygen, hypoxia induces root bending in Arabidopsis [76]. During prolonged waterlogging, low oxygen availability leads to the death of primary roots, whose function is overtaken by adventitious roots newly formed at the base of the stem closer to the surface in many plant species including tomatoes and bittersweet [77]. Low oxygen availability causes apical meristem elongation in rice induced by increased gibberellin production in a mechanism called low oxygen escape syndrome [78].

## 3. Hypoxia Influence on Soft Rot Disease

**Figure 3:**
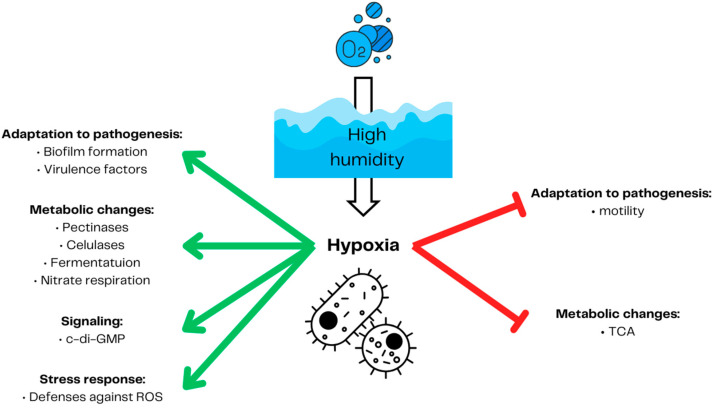
Soft rot *Pectobacteriaceae* response to hypoxia. High humidity in soil leads to decreased oxygen penetration into deeper soil. This, in turn, forces the bacteria to switch from aerobic metabolism to fermentation. Low oxygen conditions lead to the increased production of master regulator c-di-GMP. As in many other bacteria groups, hypoxia in pectinolytic bacteria leads to the activation of reactive oxygen species defenses. Pectynolytic bacteria also increase nitrate respiration production of lytic enzymes and virulence factors. Global regulator c-di-GMP favors biofilm formation versus motility, triggering soft rot *Pectobacteriaceae*’s pathogenicity. Prepared based on [89,91,92,93,94,95]. The red arrows demonstrate inhibition and the green arrows represent induction.

Hypoxia also influences plants-inhabiting beneficial and pathogenic microorganisms [79]. Although the effect of hypoxia can be beneficial, as in symbiotic *Rhizobia* and *Sinorhizobia* [14], likewise, the plant pathogenic *Plasmodiophora brassicae* [80] and *Agrobacterium tumefaciens* [81] benefit from hypoxia induced by the plant’s tumorous outgrowth. In the case of more hypoxia-sensitive organisms, such as fungi, the limited availability of oxygen may decrease the disease symptoms [6] by reducing the survival rate of hypoxia-sensitive species such as *Collybia fusipes* [22] or *Phytophthora cryptogea* [82]. Hypoxia not only influences survival but can also directly decrease the pathogenicity of some pathogen species. In *Fusarium graminearum,* hypoxia reduces the production of its main toxin, deoxynivalenol. However, it has no negative effect on its growth [23]. Also, plant susceptibility to biotic stress, such as pectinolytic bacteria (e.g., *Pectobacterium carotovorum*), can be induced by changes in plant gene expression, e.g., phenylalanine ammonia-lyase and extensions [83]. Since microbial metabolic activity in the soil can lead to root local hypoxia [84], plant pathogenic microorganisms can utilize this and compete for oxygen with their host, facilitating symptom development [80]. In this context, SRP, not only uses the decreased plant defenses [26,83,85,86] but also adapts their gene expression to hypoxic conditions to more effectively induce soft rot disease [25] (Figure 3). SRPs mainly comprise *Dickeya* and *Pectobacterium* genera and cause soft rot disease in many important vegetables and fruits [87]. *D. dadantii* [88] and *P. atrosepticum* [89] upregulate the expression of virulence factors, including pectinases and cellulases, under limited oxygen availability. These enzymes’ activity is one of the most important virulence factors for plant necrotrophic pathogens [90]. The limited oxygen environment on the rotting site of the tubers allows *P. atrosepticum* for nitrate respiration, enhancing its growth rate and invasion [91]. Pectinolytic bacteria utilize the global regulator c-di-GMP to adapt their metabolism to anaerobic or aerobic conditions [92]. A high level of c-di-GMP induced by low oxygen leads to increased virulence, biofilm formation, and decreased motility [93,94,95].

**Figure 4:**
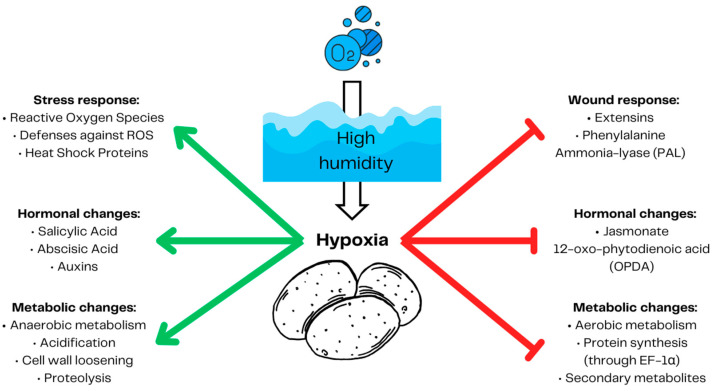
Potato tuber response to hypoxia. High humidity leads to decreased gas exchange and hypoxia. During hypoxia, potato tubers decrease aerobic respiration to preserve oxygen and increase anaerobic respiration to provide energy. Increased anaerobic respiration produces acidification and reactive oxygen species (ROS). To protect the cells from the damaging effect of ROS, plants need to activate their defenses against ROS and produce heat shock proteins. A low energetic status abolishes plant defenses, including wound response factors such as extensins, phenylalanine ammonia lyase (PAL), and secondary metabolites such as expansin (EXP), early nodulin 93 (ENOD), 4-coumarate CoA ligase-like 2 (4CL), and HMG-CoA reductase (HMG). The production of proteins is arrested by EF-1α binding to ribosomes in acidic conditions. Plant hormonal response tailored for cell elongation to escape local hypoxic conditions makes tubers prone to the attack of necrotic pathogens. Prepared based on [26,83,85,86,100,101]. The red arrows demonstrate inhibition and the green arrows represent induction.

It has been reported that hypoxia suppresses potato response to wounding (Figure 4) [26,85,86], a major route of SRP infection [96]. Potato tubers reduce anaerobic respiration to preserve oxygen. The energetic demand has to be met by induced anaerobic respiration [63]. The less effective anaerobic respiration leads to the accumulation of reactive oxygen species (ROS) and therefore requires plant upregulation of ROS defenses and the production of heat shock proteins [97]. While ROS scavengers reduce the concentration of ROS [98], heat shock proteins protect plants from harmful protein aggregates [99] emerging due to reactive oxygen species protein carbonylation [97]. Anaerobic respiration also leads to acidification, which causes elongation factor EF-1α binding to ribosomes, causing an arrest in protein production [100]. A low energetic status forces potato tubers to reduce the production of secondary metabolites and their defenses against wounding, including the production of extensines and phenylalanine ammonia lyase (PAL) [83]. Hypoxic conditions trigger plant hormonal changes, increasing the production of abscisic acid, auxins, and salicylic acid and inhibiting the production of jasmonate 12-oxo-phytodienoic acid (OPDA), which induces cell elongation to escape hypoxia and reach oxygen [101]. This, however, favors necrotrophic pathogens such as SRPs [102]. Furthermore, SRPs can cause latent infections in susceptible hosts [28] suggesting that SRPs are hemibiotrophic pathogens [31].

Even though latent infection by SRP is common and has been reported for many years [103], soft rot bacteria are considered classical necrotrophic pathogens [31]. This strange phenomenon is caused by the fact that the term necrotrophic, in contrast to biotrophic pathogens, was transferred from plant pathogenic fungi and, as such, does not describe the bacteria’s pathogenicity [104]. It has been proven that potato tubers inoculated with *Dickeya solani* initially respond with increased levels of SA, a response to the biotrophic stage, but later switch to the production of JA to target their response against the necrotrophic stage of pathogenesis [105]. This seemingly well-fitted response can easily fail when a plant is subjected to abiotic stress such as hypoxia [106]. The decreased wound response caused by hypoxia [26,86] can cause the rapid spread of pathogens and quick disease development when the plant is mechanically disrupted [107]. Even though it was proven that temperature, humidity, and inoculum are important for developing soft rot disease [37], there is still a lack of data on the transition from the latent infection of potatoes to the development of disease symptoms from both host and pathogen biology. Currently, we observe a growing number of studies dedicated to the detection of asymptomatic latent infections of potatoes with soft rot *Pectobacteriaceae* [108]. However, the subject of latent infections and the transition to symptomatic infection is still largely understudied [109,110] and, at present, the research concerning soft rot *Pectobacteriaceae* infection almost exclusively concerns symptomatic infection in disease-provocative conditions [111].

## 4. Prevention of Soft Rot Disease

### 4.1. Soft Rot Pectobacteriaceae Detection

Using certified and pathogen-free source material is still the best method of disease prevention [112]. Despite that, many farmers, especially from developing countries, use uncertified source material [113]. In Europe, where the temperature is lower and the usage of certified seed tubers is higher, the losses caused by these pathogens are estimated to be 46 M Euro yearly [27]. To reduce the huge losses caused by SRPs we can operate on the three sites of the disease triangle: the prevalence of SRPs in agricultural systems, the resistance of the potato varieties, and the influence of the environmental conditions.

Therefore, implementing modern and efficient pathogen detection methods is imperative to minimize the losses caused by plant pathogens, including SRPs [114]. The new techniques based on nucleic acids or specific antigen detection are much faster, more sensitive, and more accurate than traditional microbiology [115]. Due to developments in molecular biology, the number of available PCR-based methods for detecting SRPs is constantly increasing [108,116,117] including quantitative ones [118,119]. TaqMan PCR assays allow for specific detection of *Pectobacterium brasiliense* starting from 1000 colony forming units CFU/mL [120]. In loop-mediated isothermal amplification, it is possible to detect *P. parmentieri* with a detection limit of about 20 genomes (from gDNA isolation) or 10 CFU/mL of heat-killed bacterial cells [121]. ELISA-based methods, which could be good candidates for fast and reliable screening due to their simplicity, do not yet provide sufficient sensitivity for detecting SRPs in plant material [115]. Therefore, new methods based, for example, on immunosensors [122] or biosensors targeted for usage in the field have been created [123]. More sophisticated methods like metagenomic sequencing can be applied in seed production [124]. The early detection of SRPs can be assisted by automated image detection techniques [125,126].

Therefore, the sophisticated and sensitive methods for pathogen detection can assist seed potato producers in the production of high-quality seed material, while biosensors and visual observation-based methods can help farmers to quickly identify disease outbreaks, reducing the prevalence of SRPs in the environment.

### 4.2. Breeding for Resistance

Although early detection of pathogens can help to discard infected tubers, drastically reducing the possible losses, SRPs can overwinter in the soil, water [127], plant debris [128], or weeds [129]. Additionally, soil amendments may further decrease the chance of disease development [130]. Although, without at least partial plant resistance to the pathogens, this can lead to the rapid spread of the disease [28]. However, recent advancements in plant breeding and selection can facilitate the creation of new resistant plant cultivars [131]. The new or enhanced plant features can be obtained by cross-pollination, protoplast fusion, mutagenesis-assisted or non-assisted, agro-infection using viruses or bacteria, or transformation by foreign DNA [132]. The most time-consuming part of creating new plant varieties is selection [133]. Several generations must be developed to confirm that the new variety represents a stable and desired phenotype [131]. This process can be largely accelerated by speed breeding. Using this method, it is possible to obtain up to seven generations per year [134]. It relies, among others, on cultivating the plant in tailored photoperiod and temperature conditions to reduce the time to flowering [135]. The process can be further accelerated if we combine speed breading with genomic selection [136]. Utilizing advancements in plant physiology and genetics, high-throughput plant genomic sequencing makes an expedited process of selection possible [137]. Knowing which molecular markers are associated with resistance can facilitate the creation of new varieties with genomic selection [138]. Also, advanced phenotyping can shorten waiting for a visual phenotype to appear [139]. Firstly, we need to confirm the association between certain agronomic traits, such as resistance to pathogens with known metabolite concentration. Then, using chromatography coupled with mass spectrometry, we can verify which newly generated plant varieties possess a targeted feature before regenerating the whole plant [140]. This approach can effectively facilitate the process of selection of promising new varieties.

Although the already existing plant varieties can serve as a source of resistance against soft rot, it is suggested that wild species from the *Solanaceae* family can be a promising source of new resistance genes [141]. It has been proven that anthocyanins in colored potato varieties, that have gained popularity on the market, positively affect soft rot resistance [142]. Also, calcium has a positive influence on potato SRP resistance [143], either by soil amendment application [130] or calcium content in tubers [144,145]. It has been reported that features such as starch content [146], cell wall pectin esterification [147], and high levels of polyphenol oxidase and peroxidases [148] are positively correlated with SRP resistance. There are also indications that the genes responsible for better SRP resistance are located on the 2nd and 4th chromosomes [149]. Despite the multiple attempts to select and create new potato varieties resistant to soft rot disease [40], the prevention of this disease relies mainly on sanitation and certified seed material [30]. This is caused by the fact that the resistance to soft rot is multilocus and heavily relies on plant response to hypoxia [111]. The fact that plants belonging to the same genera differ in their response to hypoxia gives hope that new varieties more resistant to SRP can be generated [65,150]. It has been proven that, although waterlogging-susceptible potato (*Solanum tuberosum* L.) cv. Servesta and tomato (*S. lycopersicum* L. Moneymaker) activate the same ethylene signaling pathway in response to hypoxia as Bittersweet nightshade (*Solanum dulcamara* L.) (a wetland species of the *Solanum* genus) and *Arabidopsis thaliana* (L.) Heynh., this does not lead to the same hypoxia adaptations [39]. Therefore, it can be possible to transfer the waterlogging-responsive genes from wetland *Solanaceae* species to create new more resistant potato varieties. Additionally, the screening of wild native potato species can be a good source of resistance for creating new varieties [151]. The mechanisms of *Solanum microdontum* Bitter (a wild species of potato native to Bolivia) resistance are unknown, but the elevated calcium levels detected in the plant might play their part [40]. The resistance to soft rot of another wild potato species, *Solanum chacoense* Bitter, is associated with a novel mechanism of rapid wound healing [41]. Luckily, glycoalkaloid concentrations, which are toxic to humans, do not correlate with the wild potato species’ resistance to soft rot [152]. It has been shown that the offspring from the crossbreeding of two *Solanum tuberosum* Phureja Group clones can be used to breed new potato varieties with increased soft rot resistance [153]. Also, *Solanum palustre* Poepp. ex Schltdl, previously known as *Solanum brevidens* Phil., has proven to be a promising source of soft rot resistance [154,155]. The *Solanum* genus represents many species from tropical and subtropical regions adapted to high water levels and high humidity. Therefore, wild *Solanum* species can be a good source of metabolic and anatomical adaptations to waterlogging [156,157].

### 4.3. Pathogen Control

Natural plant defenses can also be stimulated by the application of chemicals [158] but also plant-beneficial microorganisms in the mechanism called induced systemic resistance (ISR) [159]. Bacteria from the species *Bacillus amyloliquefaciens* [160], *B. pumilus*, *B. subtilis*, *B. thurigiensis* [161], *B. vallismortis* [162], *Klebsiella oxytoca* [163], *Ochrobactrum lupini* [164], and *Pseudomonas chlorapis* [165] can induce plant systemic resistance against soft rot *Pectrobacteriacae*. Biological control agents (BCAs) induce plant systemic resistance by activation of Salicylic acid (SA)- or Jasmonic acid (JA)-dependent pathways [159]. It has been shown that Salicylic acid (SA) induces plant resistance to soft rot [166,167,168]. It seems counterintuitive that SA induces resistance against necrotrophic pathogens, as the defenses against those pathogens rely mainly on the Jasmonic acid (JA) pathway [102]. Indeed, the inoculation of potatoes with *Pectobacterium carotovorum* induces the JA pathway [85], but also SA in the early stages of infection [105], suggesting that SRPs are hemibiotrophic pathogens [31,104]. BCAs can also induce the natural defenses of plants against pathogens by expressing, e.g., phenylalanine ammonia-lyase (PAL) [161]. PAL is a key element of the plant’s stress response [169] to biotic and abiotic stress. Moreover, many microbial strains, including *Bacillus* spp. [170], *Pseudomonas* spp. [171], and *Trichoderma* spp. [172], produce 1-aminocyclopropane-1-carboxylate (ACC) deaminase. This enzyme, produced by many BCA targets, such as 1-Aminocyclopropane-1-carboxylic acid (ACC), is a direct ethylene precursor, alleviating the negative impact of waterlogging [173].

Moreover, most of the abovementioned bacterial strains can suppress soft rot disease by other modes of action, such as direct growth inhibition of the pathogen [160,161,162]. And indeed, it has been proven that microbial communities play a major role in potato resistance against soft rot disease [174]. The natural interactions between soil microorganisms, which may impact both pathogens and their hosts, can be considered to protect plants from soft rot [175]. There is an increasing emphasis on research dedicated to the discovery of new strains of bacteria active against soft rot disease, including, but not limited to, the genera *Agrobacterium* [176], *Bacillus* [160,176,177,178,179,180,181,182,183,184,185,186,187], *Bdellovibrio* [188], *Brevibacillus* [185], *Lactobacillus* [177], *Lellilottia* [189], *Ochrobactrum* [190,191], *Paenibacillus* [192], *Pseudomonas* [178,179,183,184,186,193,194,195,196], *Rahnella* [189], *Rhodococcus* [197,198,199], *Serratia* [110,189,200], *Streptomyces* [178,201,202], and *Variovorax* [176]. There are several reports of biocontrol strains against SRP of fungi: *Aspergillus* [203], *Penicillium* [193], and *Trichoderma* [179,181,184]; and bacteriophages: *Axomammavirus* PP1 [204]; *Cbunavirus* CB1, CB3 and CB4 [205]; *Corticovirus* PM2 [206]; *Kotilavirus* PP16 [207]; *Limestonevirus* LIMEstone1, LIMEstone2 [208]; ϕD3, ϕD5 [209]; *Myunavirus* My1 [210]; *Pemunavirus* PM1 [211]; *Phimunavirus* peat1 [212]; *Unyawovirus* DUPPII phiPccP-1 [213]; ϕPD10.3, ϕPD23.1 [214], vB_PcaM-D1, vB_PcaM-J3, vB_PcaP-A3 [215]; ZF40 [216], and others [217]. More information about bacteriophages infecting SRP has been summarized in [217,218].

The abovementioned biocontrol agents can protect plants from disease through different modes of action, such as antagonism toward pathogens, suppressing their pathogenesis, and inducing plant defenses [219]. The best-studied mechanism of action of biological control agents is the production of antibiotics [220], due to the success of antibiotics in medicine [221]. Because antibiotics have their limitations [221], other modes of action are gaining interest [219]. The fact that SRPs tightly regulate their pathogenicity and induce virulence factors in disease-favoring conditions gives them an advantage in the field but can also be used against them [222]. SRPs rely on quorum sensing to coordinate the production of virulence factors [223]. SRPs produce a range of quorum sensing N-Acyl-Homoserine Lactones: N-hexanoyl-homoserine lactone (C6-HSL), N-3-oxohexanoyl-homoserine lactone (3OC6-HSL), N-octanoyl-homoserine lactone (C8-HSL), N-3-oxo-octanoyl-homoserine lactone (3OC8-HSL), N-decanoyl-homoserine lactone (C10-HSL), and N-3-oxo-decanoyl-homoserine lactone (3OC10-HSL) [223]. While quorum sensing based on AHLs in SRPs can help these pathogens turn on their virulence when the number of bacteria is sufficient, SRPs rely on c-di-GMP hypoxia sensing [224,225]. During hypoxia, *Dickeya dadantii* activates the c-di-GMP effector VfmE, which is a quorum sensing master regulator [226]. This allows SRPs to regulate their virulence responding to environmental conditions and the pathogen population. Plants can, however, sense quorum-sensing molecules and adjust their metabolism, depending on the type of molecule, by inducing appropriate defense genes. Generally, shorter-chain AHLs induce plant resistance against necrotrophic pathogens with the use of SA signaling while longer-chain AHL induces JA signaling and resistance to biotrophic pathogens [227]. For example, 3OC8-HSL increases Arabidopsis resistance to *Pectobacterium carotovorum* by JA [228]. Moreover, it has been suggested that the mixture of different types of AHLs is the most prominent method of induction of plant defenses [229]. Additionally, many biological control agents use quorum quenching (disruption of quorum sensing) as their mode of action against soft rot disease, e.g., *Agrobacterium* [176], *Bacillus* [176], *Ochrobactrum* [190,191], *Rhodococcus* [197,198,199], and *Variovorax* [176].

Therefore, successful inhibition of soft rot can be achieved by employing various modes of action of biocontrol agents to prevent infection during the biotrophic stage and reduce pathogenicity during the necrotrophic stage [30]. Since microorganisms shape the soil structure, influencing its ability to store minerals and water [230] by producing strong osmolytes such as trehalose [231], it is worth considering if they can shape the environment to decrease the negative influence of waterlogging on oxygen availability [79]. For example, with the extended hyphal net, fungi can connect environments with different oxygen availability and perform aerobic respiration in oxygen-depleted soils and anaerobic respiration in the upper parts of the soil, shaping the bacterial soil community [232]. Filamentous fungi can alleviate waterlogging and drought stress due to their ability to transport oxygen and store water in local niches [233].

### 4.4. Shaping the Microenvironment

Moreover, protozoan predation has been proven to increase oxygen penetration in flooded rice fields [234]. The potential of microorganisms relies not only on the production of antimicrobials but on multiple co-occurring interactions between plants, microorganisms, and the environment [175]. Hence, to fully utilize their potential, a close examination of those interactions is necessary [235]. However, while microorganisms can shape the microenvironment, they not only influence the plant and pathogens but also each other. Unfortunately, not much information has been published on the subject of the environmental impact on the activity of BCAs [79]. Waterlogging harms oxygen availability for microorganisms due to lower oxygen diffusion and competition with the host [236]. Plants decrease oxygen loss through the roots by their adaptations to flooding [5]. Reduced oxygen availability influences the microbial composition of soil favoring anaerobic over aerobic microorganisms [237]. This can harm the plant because of the negative influence on the abundance of microorganisms from plant-beneficial taxa (such as *Streptomyces*), which depend on oxygen supplementation by the plant roots. Also, the population of microbial endophytes and arbuscular mycorrhiza is significantly reduced by flooding [238]. However, there are some plant-beneficial endophytes including bacteria from the phyla: *Deltaproteobacteria*, *Firmicutes* [237], and *Aquapirillium* (*Proteobacteria*) [239], and fungi, mainly dark septate endophytes (DSE) [240], arbuscular mycorrhizal fungi (AMF) [241], and yeast: *Cryptococcus*, *Exophiala*, *Sporobolomyces*, and *Rhodorotorula* [242], which can adapt to anaerobic conditions. Microbial communities and their metabolism may also be influenced indirectly by waterlogging due to changes in plant exudation. In some cases, plants can implement mechanisms called “cry for help” which involve increased root exudation to attract plant-beneficial microorganisms [243]. On the other hand, the plant’s anaerobic metabolism creates products that are exudated to minimize their toxicity for the plant [244]. However, these toxic products (e.g., ethanol) can act as chemoattractants for pathogens sensing the host in a poor metabolic state [245]. Although we have some knowledge of how hypoxia influences the plant microbial communities [79], and its influence on the plant pathogen metabolism [6], very little is known about its influence on plant-beneficial microorganisms. It is suggested that the application of beneficial PGPR can only be successful when the soil is properly aerated [246]. Fighting against pathogens well adapted to hypoxic conditions with bacteriophages that do not affect their metabolism could be considered a method of choice in these circumstances. Unfortunately, the influence of hypoxia on the activity of bacteriophages is largely understudied [247,248]. For example, *Pseudomonas aeruginosa* tends to develop resistance to the myovirus PAK_P1 infection [249]. However, in the case of *Salmonella* s25pp, hypoxia reduces the infection efficiency and the burst size of the ϕSan23 bacteriophage but also decreases the number of resistant mutants [248]. Unfortunately, the data on the influence of hypoxia on plant pathogens and bacteriophages are lacking.

## 5. Future Perspectives

Due to climate change [38] and global vegetable transport, we observe rising losses caused by soft rot disease [250]. Knowledge on the pathogenicity of soft rot *Pectobacteriace* is constantly growing, especially owing to new advanced molecular methods [31]. Our understanding of plant responses to hypoxia has also recently been furthered significantly [251]. However, there remains a gap in the research encompassing environment, pathogens, and plant responses [31]. A comprehensive approach, including various strategies, such as good agricultural practices, sanitation, usage of disease-free seed material, early pathogen detection, breeding resistant varieties, and targeted disease control, is required to protect crops from bacterial diseases [252]. However, we need to put great emphasis on the involvement of environmental conditions which are crucial for the development of the disease. Potatoes and SRPs respond differently to waterlogging and the resulting hypoxia, which has a strong influence on all approaches for the mitigation of soft rot disease (Table 1).

Due to the high prevalence of SRPs in the environment and not fully established sources of infection, the chance of infection during potato production is high [31]. This calls for the development of sensitive and reliable methods for SRP detection during seed tuber production. Additionally, the population dynamics of different SRP species require adjustment of the used methods to the currently most threatening species from this group [87]. While sophisticated and sensitive methods are best suited for the process of seed tuber certification, the development of new, fast, and simple methods of detection (e.g., immunoenzymatic techniques) are crucial for the rapid identification and isolation of SRP foci of infection [122].

However, the usage of pathogen-free seed material and rapid disease identification in the field can help to reduce the pathogen load. The high incidence of soft rot disease during years with high precipitation requires the creation of new more resistant potato varieties. Breeding new resistant varieties and biological plant protection are considered the most promising approaches to fighting soft rot disease [112]. The use of the CRISPR-Cas system enables the easy and fast creation of new varieties of plants and has been gaining social acceptance for its application in agriculture [253]. Using wild *Solanum* species as a source of new genes in potato breeding seems promising for obtaining new varieties that are more resistant to diseases [254]. The majority of *Solanum* plants are native to tropical regions and possess multiple metabolic and physiological adaptations to high humidity [156,157]. The *Solanum* genus encompasses many wild potato species that possess promising strategies to mitigate waterlogging stress [151]. Therefore, the creation of new potato varieties less prone to hypoxia during waterlogging can help to alleviate the environmental factor favorable for the development of soft rot disease.

The resistance of crops to hypoxia and soft rot can be further increased by favorable microbiota. Biological plant protection can be a valuable source of resistance against SRPs [255] and mitigation [159] of waterlogging stress, which favors SRP pathogenesis [83]. Many new biocontrol bacteria strains and bacteriophages [256] against SRPs have been isolated recently [112]. Combining those isolates in multispecies consortia can be a promising approach to disease protection in changing environmental conditions [257]. However, it is important to consider the influence of hypoxia on their activity and how they can alleviate this abiotic stress, to decrease the chance of disease development [4]. Biological plant protection agents against soft rot disease should remain active in hypoxic conditions and not be outcompeted by the pathogens when the oxygen availability is lowered. Additionally, new biological control stains should be tested for their ability to decrease potato resistance to waterlogging, e.g., by inducing their systemic resistance [166,167,168] or via production of 1-aminocyclopropane-1-carboxylate (ACC) deaminase [170,171,172]. The ability of microorganisms to shape the soil structure can also be considered a promising method for the alleviation of waterlogging stress, which decreases the chance of the development of soft rot disease [230].

To thoroughly plan an integrated approach of soft rot control, we must combine current knowledge in molecular biology, plant physiology, and pathogen biology, especially in the presence of coexisting abiotic stress [2]. This task requires tighter cooperation between scientists representing those fields [252].

## Figures and Tables

**Table 1:** Influence of waterlogging and subsequent hypoxia on soft rot *Pectobacteriaceae* and potato tubers (*Solanum tuberosum* L.) in relation to the development of soft rot disease. The summary from Figure 3 and Figure 4.

Hypoxia Influence on SRPs and Potato		
	Positive	Negative
Soft Rot *Pectobacteriaceae*	**Adaptation to pathogenesis:** Biofilm formation Virulence factors **Metabolic changes:** Pectinases Cellulases Fermentation Nitrate Respiration **Signaling:** c-di-GMP **Stress response:** Defenses against ROS	**Adaptation to pathogenesis:** Motility **Metabolic changes:** TCA
Potato (*Solanum tuberosum* L.)	**Stress response:** Reactive Oxygen Species Defenses against ROS Heat Shock Proteins **Hormonal changes:** Salicylic Acid Abscisic Acid Auxins **Metabolic changes:** Anaerobic metabolism Acidification Cell wall loosening Proteolysis	**Wound response:** Extensins Phenylalanine Ammonia-lyase (PAL) Hormonal changes: Jasmonate 12-oxo-phytodienoic acid (OPDA) **Metabolic changes:** Aerobic metabolism Protein synthesis (through EF-1α) Secondary metabolites

## Data Availability

Data sharing is not applicable.
